# Follicular Lymphoma of the Breast With Secondary Central Nervous System Transformation and Concurrent Metastatic Melanoma: Report of a Rare Case

**DOI:** 10.7759/cureus.93232

**Published:** 2025-09-25

**Authors:** Pranesh Gavali, Vijay S Kollipara, Shravani Rama

**Affiliations:** 1 Internal Medicine, Seth Gordhandas Sunderdas Medical College, Mumbai, IND; 2 Internal Medicine, Gandhi Medical College, Hyderabad, IND; 3 Obstetrics and Gynecology, Gandhi Hospital, Hyderabad, IND

**Keywords:** breast lymphoma, cns lymphoma, dual malignancies, extranodal diffuse large b-cell lymphoma, individualized therapy

## Abstract

Follicular lymphoma (FL) rarely presents in the breast and even less frequently transforms with secondary central nervous system (CNS) involvement. The coexistence of transformed FL with metastatic melanoma is exceptionally rare and presents significant diagnostic and therapeutic challenges. We report the case of an elderly female initially diagnosed with primary breast follicular lymphoma (FL), managed with surveillance. Over subsequent years, she experienced multiple relapses, treated with bendamustine-rituximab and later lenalidomide, achieving remission. Fourteen years after initial diagnosis, she developed CNS symptoms with cerebellar lesions; resection revealed transformed diffuse large B-cell lymphoma (DLBCL) of germinal center origin (CD5+, CD10+, BCL2, BCL6, MYC+, Ki-67 ~70%). Concurrent metastatic melanoma was also diagnosed. Given the rarity of CNS-transformed FL and the absence of standard guidelines, her case was managed as primary CNS lymphoma. She received high-dose methotrexate, rituximab, and temozolomide with Stereotactic Body Radiotherapy (SBRT) for melanoma. Despite significant intracranial progression, she remained largely asymptomatic and prioritized quality of life, highlighting the challenges of individualized care in complex dual malignancies.

## Introduction

Follicular lymphoma (FL) is an indolent B-cell non-Hodgkin lymphoma that accounts for approximately 20-30% of all non-Hodgkin lymphomas, most often presenting with widespread nodal disease in older adults [1]. Although initial clinical behavior is typically slow, histologic transformation to an aggressive subtype, most commonly diffuse large B-cell lymphoma (DLBCL), occurs at a rate of about 2-3% per year and significantly worsens prognosis [2,3]. Central nervous system (CNS) involvement in FL is extremely rare, with an estimated incidence of less than 1% [4,5]. Additionally, transformation of FL recurrence in the brain to DLBCL is very rare, with only nine reported cases worldwide. Moreover, five out of nine patients died within 2.5 years, indicating a poorer prognosis [6].

Management of CNS involvement due to transformed FL is particularly challenging due to the lack of consensus guidelines, and treatment is often extrapolated from protocols used in primary CNS lymphoma, involving high-dose methotrexate, rituximab, and temozolomide [7,8]. Here, we present a rare case of FL initially presenting in the breast, with multiple relapses over 15 years, culminating in CNS transformation and concurrent metastatic melanoma. The clinical course highlights the complexity of management in the absence of standardized treatment pathways and highlights the need for individualized, multidisciplinary care in this rare occurrence.

## Case presentation

A 76-year-old Caucasian female with past medical history of hypertension and hyperlipidemia presented following an abnormal screening mammogram. Initial suspicion was for primary breast carcinoma; however, stereotactic biopsy revealed an extranodal non-Hodgkin lymphoma, specifically grade I follicular lymphoma. Pathology was confirmed upon external review. Staging investigations demonstrated no evidence of disease elsewhere. Most of the visible diagnosed tissue was surgically removed, and she underwent surveillance treatment. A year later, during follow-up, the patient presented with a palpable right axillary mass. Excisional biopsy revealed grade II follicular lymphoma, and systemic staging done by PET-CT showed no disease elsewhere. She was continued under observation.

Nine years later, she presented with fatigue, anorexia, and weight loss that began a month ago. CT of the abdomen and pelvis revealed prominent mesenteric adenopathy. Bone marrow biopsy showed B-cell lymphoma, but immunophenotyping was suggestive of lymphoplasmacytic lymphoma or marginal zone lymphoma. PET-CT confirmed extensive lymphadenopathy involving the central mesentery and retroperitoneum, along with multifocal extranodal involvement. The patient underwent six cycles of bendamustine and rituximab, resulting in a marked response on post-treatment PET-CT scan.

At a subsequent follow-up 2 years later, new left axillary lymphadenopathy was noted. PET-CT revealed widespread lymphadenopathy above and below the diaphragm, involving multiple nodal stations as well as hepatic and splenic uptake, though without any evidence of macroscopic bone marrow involvement. Four cycles of salvage therapy with lenalidomide were initiated for relapsed follicular lymphoma. PET-CT after completion of therapy showed a complete metabolic response to the treatment.

**Figure 1:**
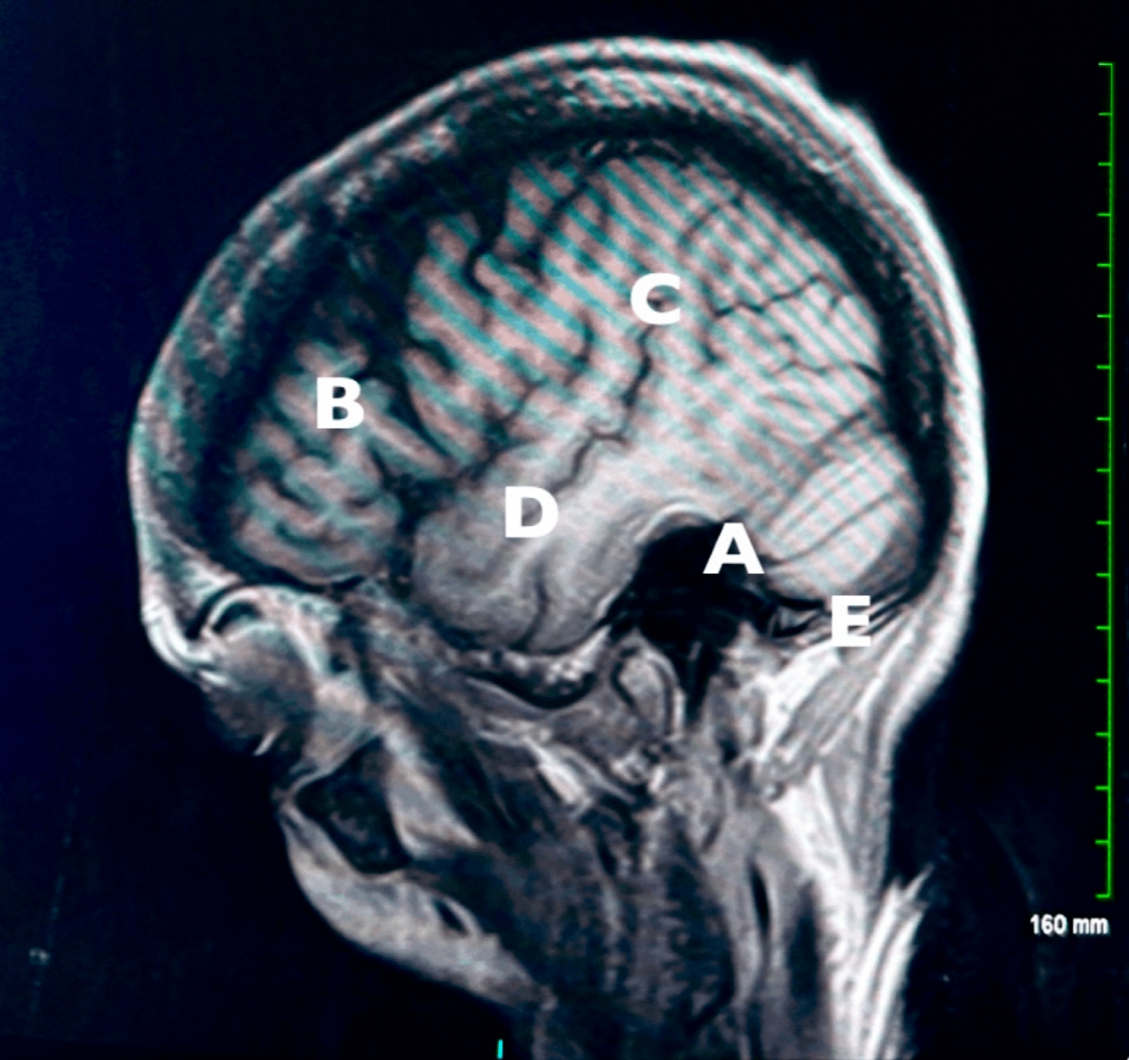
Sagittal MRI of the brain demonstrating suspected stage 4 metastasis of follicular lymphoma transformed to DLBCL in the left cerebellar hemisphere The lesion is seen in the occipital lobe (A). Labeled structures include the frontal lobe (B), parietal lobe (C), temporal lobe (D), and cerebellum (E). DLBCL: diffuse large B-cell lymphoma

Four years later, during routine surveillance, PET-CT revealed a hypermetabolic focus in the left occipital lobe. The patient also complained of ataxia and dizziness at that time. A subsequent brain MRI showed lesions measuring 9 mm and 5 mm in the left cerebellar hemisphere with surrounding edema and additional satellite lesions laterally with no significant mass effect on the 4th ventricle, as shown in Figure 1. The patient underwent craniotomy with resection of the cerebellar mass. Histopathology demonstrated CD5-positive follicular lymphoma with a high Ki-67 proliferation index (70%) and additional features on immunohistochemistry. Further review at a tertiary center identified CD10 subset, BCL2, BCL6, and MYC positivity. These findings demonstrated an aggressive large B-cell lymphoma with a germinal center that may be consistent with histologically large cell transformation of underlying follicular lymphoma. It is extremely rare to see CNS involvement with follicular lymphoma, and moreover, transformed diffuse large B-cell lymphoma.

Given the rarity of CNS involvement in the recurrence of follicular lymphoma, treatment options were limited due to the absence of clear guidelines on the management of this recurrence. A discussion with lymphoma experts concluded that this be treated as a primary CNS lymphoma. On those grounds, high-dose methotrexate in combination with rituximab and temozolomide was considered, but it posed significant risks due to the patient's age and comorbidities. Complicating the clinical course, the patient also had metastatic melanoma diagnosed a year ago, with a pulmonary nodule identified on imaging and confirmed via biopsy. She underwent stereotactic body radiotherapy for melanoma while lymphoma-directed therapy was temporarily deferred.

However, subsequent brain imaging demonstrated significant progression of CNS disease, with enlargement of cerebellar lesions, new enhancing masses in the frontal lobe, and diffuse involvement of the corpus callosum and bilateral parietal lobes. Even though there was significant progression of the intracranial lymphoma, it was of much surprise that she did not have any symptoms. Treatment with high-dose methotrexate combined with rituximab and temozolomide was started. In view of her advanced age, methotrexate 3 g/m2 with leucovorin rescue was recommended. Considering the side effects, hospital admission was necessary for the administration of high-dose methotrexate. After discussion with the Radiation Oncologist, it was mutually decided to start treatment cycles for CNS lymphoma and Stereotactic Body Radiation Therapy (SBRT) for metastatic melanoma to be given between the treatment cycles. The patient currently has no significant symptoms and is planning to enjoy her life as much as she can, and in view of that, she is currently in Florida enjoying her vacation.

## Discussion

Follicular lymphoma (FL) is an indolent B-cell subtype of mature B-cell non-Hodgkin lymphoma, typically presenting with nodal disease. Extranodal involvement of the breast is exceedingly rare. Primary breast lymphoma accounts for only 0.4-0.5% of breast malignancies and about 2% of extranodal NHLs, with follicular lymphoma (FL) being far less common than diffuse large B-cell lymphoma (DLBCL) [9]. When FL presents as a localized extranodal lesion without systemic disease, management options include radiotherapy, observation, or excisional biopsy, serving both diagnostic and therapeutic purposes [10]. In this patient, stereotactic biopsy followed by surveillance was appropriate, given her age, comorbidities, and limited disease. Subsequent detection of grade II FL in the axilla illustrates FL’s tendency for localized recurrence and supports continued observation [11].

Lymphoplasmacytic lymphoma (LPL) and marginal zone lymphoma (MZL) can infiltrate bone marrow similarly, relying on plasmacytic differentiation and pattern recognition for differentiation. Paratrabecular and interstitial infiltration are more frequent in LPL, whereas MZL often shows intrasinusoidal and nodular patterns [12]. Mesenteric and retroperitoneal adenopathy with extranodal spread is more characteristic of extranodal or nodal MZL than LPL. Extranodal MZL can involve the gastrointestinal (GI) tract, mesentery, and bone marrow in up to 30-50% of cases [13].

Bendamustine-rituximab (BR) is highly effective and well-tolerated in first-line treatment for indolent lymphomas like MZL and LPL. Marked imaging response post-BR supports the diagnosis of an indolent lymphoma subtype, rather than aggressive transformation [14]. FDG uptake in MZL is highly variable. Some lesions may go undetected, necessitating confirmation with CT or MRI. PET-CT excels at suggesting but not confirming transformation. Biopsy remains the diagnostic gold standard [15]. Long-term survival in MZL varies by subtype. Mucosa-associated lymphoid tissue​​ (MALT) lymphoma has the best outcomes, while splenic and nodal MZL show slightly lower survival but still a favorable prognosis [16].

The finding of widespread lymphadenopathy above and below the diaphragm, along with hepatic and splenic uptake, is characteristic of advanced-stage FL. Bone marrow (BM) sparing is less common in advanced FL (BM involvement occurs in ~70% at diagnosis) but does not rule out active systemic disease [17]. R2 (rituximab + lenalidomide) is a non-chemotherapy option with proven efficacy in relapsed FL. The efficacy of R2 compared with rituximab alone in an AUGMENT trial is mentioned in Table 1 [18].

**Table 1:** Efficacy of Lenalidomide + Rituximab (R²) in Relapsed/Refractory FL and MZL - AUGMENT Trial The information in this table has been created by the authors from the Phase III AUGMENT trial paper [18] and not exactly reproduced from the article. FL: follicular lymphoma; MZL: marginal zone lymphoma; CMR: complete metabolic response

Category	R² (Lenalidomide + Rituximab)	Rituximab Alone
Mechanism of Action	Enhances immune-mediated killing; counters tumor microenvironment suppression; synergistic with rituximab	CD20-directed monoclonal antibody
Population	Relapsed/Refractory FL and MZL	Relapsed/Refractory FL and MZL
Progression-Free Survival (PFS)	39.4 months	14.1 months
Overall Response Rate (ORR)	78%	53%
Complete Response (CR) Rate	34%	18%
5-Year Overall Survival (OS)	~83%	~77%
Clinical Implication	Durable PFS and CMR associated with long-term disease control	Less durable response

Secondary CNS relapse in FL is rare, which is reported in only ~9-10 cases over decades and typically involves both cerebral and cerebellar parenchyma [6]. Despite its indolent nature, FL can infiltrate the CNS late in its course, often without histologic transformation. MRI typically reveals multiple homogeneously enhancing parenchymal lesions and perilesional edema. Ring-enhancing or satellite lesions are common in secondary CNS lymphoma. Differentiating from metastases or glioma relies on lesion distribution and PET-CT correlation [19]. Several cases with cerebellar relapse have been reported.

Historically, prognosis has been poor with approximately five of the nine reported cases passing away within 2.5 years post-CNS relapse despite aggressive multi-modality therapy [6]. New-onset neurologic signs such as ataxia and dizziness indicate posterior fossa involvement. Together with PET-CT and MRI findings (9 mm and 5 mm cerebellar lesions with edema), these strongly suggest CNS relapse [13,20]. This patient’s widespread systemic disease hints at increased CNS relapse risk; future cases might warrant proactive CNS-directed surveillance or prophylaxis [21]. Brain PET-CT can detect hypermetabolic foci correlating with MRI findings and uncover systemic disease. Fluorodeoxyglucose-Positron Emission Tomography (FDG-PET) aids both diagnosis and comprehensive staging in suspected cases of secondary CNS lymphoma [19].

Follicular lymphoma rarely transforms into aggressive large B-cell lymphoma (DLBCL) involving the CNS. Both CNS involvement and histologic transformation are individually uncommon and even more so together [20]. The presence of transformed disease in the cerebellum following resection underscores the aggressive clinical shift [20]. CD5 positivity in FL is rare and has been associated with a more aggressive biology, a higher risk of transformation, and poorer prognosis [22,23]. Co-expression of CD5 and CD10, often observed during transformation to high-grade lymphoma, suggests clonal evolution and acquired phenotypic shifts [24].

The transformed tumor expressing CD10, BCL2, BCL6, and MYC with high Ki-67 (~70%) is consistent with double-expressor or double-hit DLBCL, associated with rapid proliferation and transformation from FL [25]. High Ki-67 index further supports aggressive tumor kinetics [25]. Transformed DLBCL consistently shares clonal markers (e.g., IGH-BCL2 rearrangement) with antecedent FL [23]. CD5 expression may emerge during transformation, reflecting genetic evolution and disease progression [24].

Central nervous system involvement in secondary DLBCL is uncommon and portends poor outcomes; double-hit or double-expressor profiles further increase the risk of CNS invasion [26]. The cerebellar presentation with multiple satellite lesions reflects biologic aggressiveness typically seen in transformed high-grade lymphomas [26]. Given the transformed immunophenotype and molecular risk (MYC/BCL6, high Ki-67), aggressive combination therapy similar to other double-hit lymphomas is warranted [25]. However, early surgical resection and prompt initiation of CNS-directed systemic therapy may improve outcomes in isolated lesions [27].

The decision to treat this case as primary CNS lymphoma reflects current clinical practice when faced with CNS involvement by systemic lymphomas [21]. High-dose methotrexate (HD-MTX) is the cornerstone of CNS lymphoma treatment, as it is the most effective agent capable of crossing the blood-brain barrier and achieving cytotoxic concentrations in the cerebrospinal fluid [28]. The proposed combination of HD-MTX with rituximab and temozolomide (the RTM regimen) has demonstrated efficacy in both primary and secondary CNS lymphoma, with overall response rates of 67-81% in various studies [29,30,31]. The addition of rituximab to methotrexate-based regimens improves treatment response, particularly when combined with other blood-brain barrier-penetrating agents like temozolomide [30].

The patient’s advanced age and comorbidities significantly influenced treatment decision-making, as elderly patients with CNS lymphoma face substantially higher treatment-related risks [31]. The 5-year survival rate for all treated patients is only 31%, with outcomes being particularly dismal in the elderly population [28]. HD-MTX in elderly patients requires careful dose adjustments based on creatinine clearance, as declining renal function can lead to drug accumulation and increased toxicity [31].

The presence of concurrent metastatic melanoma added another layer of complexity. Stereotactic body radiotherapy (SBRT) represents an effective local treatment option for oligometastatic melanoma, offering excellent local control (90-94% at 1-3 years) with minimal toxicity [32]. Prioritizing SBRT for the melanoma metastasis while deferring lymphoma-directed therapy is a reasonable approach in medically complex patients [32].

Despite substantial radiologic disease progression like enlargement of cerebellar masses, new frontal lesions, and diffuse involvement of the corpus callosum and parietal lobe, the patient remained remarkably asymptomatic. Asymptomatic radiologic progression is unusual but has been reported in CNS lymphoma, emphasizing the unpredictability of manifestations and the importance of routine imaging [33]. HD-MTX remains the mainstay of induction therapy due to its ability to penetrate the blood-brain barrier and achieve cytotoxic CNS concentrations [34]. The addition of rituximab and temozolomide is supported by emerging evidence suggesting synergistic effects and tolerable toxicity in older adults [35,30,36].

For her pulmonary melanoma metastasis, the team elected to use stereotactic radiotherapy (SBRT) between lymphoma treatment cycles. SBRT is an established, effective approach to lung-confined oligometastatic melanoma, offering high local control with favorable toxicity, which is critical for patients with limited systemic treatment options or diminished tolerance [37]. Despite ongoing simultaneous treatments, the patient exhibited resilience and optimism, even planning a vacation to Florida. Evidence suggests such positive outlooks can improve quality of life, reduce distress, and support treatment adherence in cancer patients [38,39].

## Conclusions

This case highlights the clinical complexity of follicular lymphoma (FL) arising in the breast with subsequent transformation and central nervous system (CNS) relapse. Although primary breast FL is rare, its potential for local recurrence, transformation into diffuse large B-cell lymphoma (DLBCL), and late CNS involvement illustrates the heterogeneous disease course. Treatment decisions are further complicated by advanced age, comorbidities, and competing malignancies, often requiring a tailored approach that balances efficacy with tolerability.

Importantly, no standardized guidelines exist for the management of secondary CNS lymphoma from recurrent FL, leading clinicians to adopt treatment strategies established for primary CNS lymphoma. This case highlights the need to develop evidence-based guidelines addressing secondary CNS involvement, with emphasis on risk stratification, CNS surveillance, and tolerable regimens for elderly patients. Future research should also evaluate molecular predictors of transformation and the role of radiotherapy in patients with dual malignancies. Establishing such frameworks may ultimately improve outcomes in these rare, high-risk presentations. Equally, the optimistic outlook of our patient highlights the importance of integrating psycho-oncology into care, as resilience and positivity can enhance quality of life and complement clinical management.

This case also highlights the miraculous ability of human beings to live to the fullest, even when nature has cunning ways of finding our weakest spots.
